# News and Notes

**Published:** 1901-05

**Authors:** 


### *NEWS AND NOTES.*

New Orleans Polyclinic—Special Notice.—On account of various requests the session of the Polyclinic will be continued to May 31, instead of May 11th, as announced in catalogue. Isadore Dyer, M. D., Secretary. P. O. Box 797, New Orleans, La.

---

The Board of Governors of the Pasteur Institute Laboratory met in Atlanta, April 12th, to receive the first semi-annual report of the physician, Dr. Jas. N. Brawner, and the pathologist, Dr. Claude A. Smith. Since December 1st eleven cases have received the Pasteur treatment, and all dismissed as cured. Eight were bitten by rabid dogs as proved by inoculation of rabbits. The pathological department is doing good work also, and the Board was highly pleased with the first report.

---

The Twenty-sixth Annual Meeting of the American Academy of Medicine will be held at the Hotel Aberdeen, St. Paul, Minn., on Saturday, June 1, 1901, at 11 A.M. (Exécutive Session: the Open Session beginning at 12 M.), continuing through Monday, June 3d.

The principal features of the meeting will be a symposium on "Institutionalism," and another on "Reciprocity in Medical Licensure." Series of valuable papers on both topics have been promised, as well as interesting papers on some other subjects. The President's Address (Dr. S. D. Risley, of Philadelphia, will be delivered on Saturday evening, June 1st, and the Annual Social Session held on Monday evening, June 3d.

Members of the profession are always welcomed to the open sessions of the Academy. The Secretary (Dr. Charles McIntire,

Easton, Pa.) will be pleased to send the program, when issued, blank applications for fellowship, etc., when requested to do so.

---

DR. WM. JAY YOUMANS, for many years editor of *Popular Science Monthly,* to-day died at his home, in Mt. Vernon, N. Y., a suburb of this city, of typhoid fever, after an illness of ten days. When difficulties a year ago came upon the house of Appleton, and the *Science Monthly* was transferred, Dr. Youmans severed his connection with it, and retired permanently from active life. He was deeply attached to a handsome farm possessed by him among the hills near Saratoga, N. Y., where he was born, October 14, 1838, and near which he began his education in a district school. Dr. Youmans studied chemistry with his brother, Edward Livingston Youmans, at Columbia College and at Yale Scientific School. He graduated in medicine from New York University in 1865.

---

THE ST. PAUL MEETING AND YELLOWSTONE PARK.—Arrangements have been completed for an excursion of the members of the American Medical Association to Yellowstone Park. The Committee of Arrangements has finally succeeded in persuading the officials to open up the park a week earlier than usual in order to accommodate the Association. A special train will be run from St. Paul to the Yellowstone Park and the railroad officials have promised to do everything in their power to make it satisfactory to all concerned. The rates will be very low, but how low can not at this time be definitely stated. Those who attended the meeting in 1882 will remember with much pleasure a similar excursion that was run at that time, and these will not need to be informed that the one now proposed will be full of enjoyment. Further announcements will be made later. The Yellowstone National Park contains more natural wonders than are to be found anywhere else in the world, and this will be a rare opportunity for our Eastern friends to see what this portion of our Great West possesses.

